# Clinical Implementation of a High-Throughput Automated Comprehensive Genomic Profiling Test: TruSight Oncology 500 HT

**DOI:** 10.1016/j.jmoldx.2024.11.005

**Published:** 2024-12-12

**Authors:** Markus Ball, Eva Romanovsky, Fabian Schnecko, Martina Kirchner, Olaf Neumann, Regine Brandt, Susanne Beck, Huriye Seker-Cin, Klaus Kluck, Iordanis Ourailidis, Hannah Goldschmid, Annette Fink, Anna-Lena Volckmar, Michael Menzel, Michael Allgäuer, Peter Schirmacher, Jan Budczies, Albrecht Stenzinger, Daniel Kazdal

**Affiliations:** ∗ Institute of Pathology, Heidelberg University Hospital, Heidelberg, Germany; † Translational Lung Research Center (TLRC) Heidelberg, Member of the German Center for Lung Research (DZL), Heidelberg, Germany; ‡ Center for Personalized Medicine Heidelberg, Heidelberg, Germany; § German Cancer Consortium, Heidelberg, Germany

## Abstract

The adoption of comprehensive genomic profiling in oncology has rapidly increased the demand for standardized tumor sample processing in diagnostic laboratories. Automation of DNA and RNA library preparation workflows offers the possibility to scale-up and standardize sample processing. We report on the clinical implementation of the automated TruSight Oncology 500 High-Throughput library preparation workflow from formalin-fixed, paraffin-embedded tumor samples using the Biomek i7 hybrid Workstation. Using the same input amount, the automated workflow was validated against manual library preparation. Quality control metrics (total and mapped reads, median insert size, and median exon coverage) and the detection of tumor mutational burden, a complex biomarker, were concordant between the manual and automated workflows. The automated workflow was implemented on a total of 2997 pan-cancer clinical samples to detect genomic variants and complex biomarkers. Workflow automation resulted in a 4-fold reduction in hands-on time and a 1.7-fold reduction in total runtime compared with manual library preparation (6 hours vs. 23 hours; 24 hours vs. 42.5 hours, respectively) for a 48 DNA + 48 RNA sample batch. The automated workflow required one technician versus three technicians to manually prepare the same number of libraries. This study shows that implementation of the automated TruSight Oncology 500 High-Throughput workflow significantly reduced hands-on time and processing time per sample compared with manual library preparation.

The adoption of comprehensive genomic profiling (CGP) through next-generation sequencing in oncology is fundamental for patient access to targeted cancer therapy.^1^, ^2^, ^3^ Patient outcomes are linked, in part, to timely access to targeted therapy, which is highly dependent on turnaround time of CGP results.^4^, ^5^, ^6^, ^7^ Availability of genomic sequencing results before first-line therapy was shown to be associated with significantly better overall survival in patients with metastatic non–small-cell lung cancer.^4^ Conversely, treatment initiation before CGP results are available can negatively affect patient outcomes.^7^ Therefore, a short turnaround time is crucial for adequate patient care. Globally, median next-generation sequencing turnaround times vary between 18 and 21 days.^8^^,^^9^ A recent survey conducted among cancer care professionals in France, Germany, Italy, and Spain reported that in-house next-generation sequencing solutions were generally considered clinically superior and faster.^10^ Diagnostic laboratories can accelerate sample processing by optimizing CGP workflows. A strategy to shorten turnaround time is to automate DNA and RNA library preparation workflows in combination with high-throughput sequencing capabilities.^11^^,^^12^

The current article describes automation of the TruSight Oncology 500 (TSO 500) DNA High Throughput (HT) assay (Illumina, San Diego, CA) using the Biomek i7 Hybrid (hyb) Workstation (Beckman Coulter, Brea, CA) compared with the manual TSO 500 DNA assay. Also included are the first automated TSO 500 RNA HT results from formalin-fixed, paraffin-embedded (FFPE) samples. The TSO 500 HT assay interrogates 523 cancer-relevant genes from DNA and 55 genes from RNA, plus microsatellite instability (MSI) and tumor mutational burden (TMB), with the flexibility to batch up to 192 samples per flow cell. This article reports on the implementation and diagnostic performance of the automated TSO 500 DNA HT assay to identify variants of clinical significance, as well as TMB and MSI. This study shows the successful implementation and validation of an automated CGP approach for clinical FFPE samples applying the TSO 500 DNA HT workflow in combination with the Biomek i7 platform. Furthermore, comparison of the first 2997 automated samples representing >30 tumor types processed is presented, and the performance with a sample set of 2876 manually prepared libraries regarding quality metrics of the libraries, scalability, and technician hands-on time for different batch sizes is evaluated.

## Materials and Methods

### Samples

#### Validation Cohort

For validation of the automated workflow, a cohort of 73 clinical samples were analyzed by using the manual TSO 500 DNA workflow and the automated TSO 500 DNA HT workflow. Runs were compared for library performance data, including percentage of aligned reads, median insert size, and median family size, describing the mean PCR duplicate reads collapsed to a consensus read, and median exon coverage. The diagnostic biomarkers of mutation calls, TMB, and percentage of microsatellite unstable sites were also evaluated.

#### Clinical Cohorts

For a comprehensive comparison between both approaches, the distribution of quality control parameters and mutation prevalence was compared in two distinct clinical cohorts that were analyzed by using the manual TSO 500 DNA or automated TSO 500 DNA HT (both Illumina) approach, respectively. A total of 5873 FFPE diagnostic tissue samples of several tumor types underwent comprehensive molecular workup at the Institute of Pathology of the University Hospital Heidelberg (Heidelberg, Germany). The TSO 500 DNA panel was used for the manual workflow and included 2876 samples that were analyzed between April 23, 2018, and January 1, 2021. The TSO 500 DNA/RNA HT library kit was used for the automated workflow for 2997 samples from September 29, 2021, to January 8, 2024. Sample and data processing protocols were approved by the ethics committee of Heidelberg University (S-315/2020).

### DNA/RNA Extraction and Quantification

Tumor areas were marked on an hematoxylin and eosin–stained slide, and corresponding tissue areas were macrodissected from subsequent unstained slides. Tumor cell content was estimated by an experienced pathologist using a microscope.

DNA and RNA were extracted automatically using 5- to 10-μm thick FFPE sections as input and applying the Maxwell RSC Benchtop Instruments with the Maxwell RSC DNA and/or RNA FFPE Kit (Promega, Madison, WI), in accordance with the manufacturer’s instructions. Total DNA and RNA concentrations were determined by using the Qubit HS DNA assay and Qubit HS RNA assay (Thermo Fisher Scientific, Waltham, MA), according to the manufacturer’s protocols.

### Library Preparation and Sequencing

DNA integrity was assessed by using the Genomic DNA ScreenTape Analysis on a 4200 TapeStation System (both Agilent, Santa Clara, CA). Up to 80 ng DNA was fragmented, targeting a mean fragment size around 200 bp, according to the manufacturer’s protocol, using the ultrasonicators ME220 or ML230 (Covaris, Woburn, MA). Libraries were prepared either manually, using the TSO500 DNA Kit according to the manufacturer’s protocol, or via an automated procedure using the TSO 500 HT library kit (Illumina) on a Biomek i7 hyb liquid handler (Beckman Coulter) with an Illumina qualified workflow TSO 500 HT Automated Workflow on the Biomek i7 Method Setup Guide 21.07.2206 (Beckman Coulter). TSO 500 DNA and TSO 500 DNA HT libraries were sequenced with a target of 40 to 50 million read pairs per sample, TSO 500 RNA HT libraries with 10 million read pairs, independent of the sequencing platform targeting approximately 1000× mean coverage per sample in target regions, using either 300 cycle high-output kits on a NextSeq 500 or SP or S1 200 cycle V1.5 kits on a NovaSeq 6000 system (both Illumina). Sequencing data were processed using the Dockers TruSight Oncology 500 v2.2 Local App (automated workflow) and TruSight Oncology 500 v1.3.0.32 Local App (manual workflow).

The primary differences between the manual and automated workflows included the following: target DNA peak fragment size was reduced for ultrasonication from 250 bp (manual) to 200 bp (automated), the TSO 500 Docker pipeline was updated from local app version 1.3.0.32 to version 2.2, and the libraries from the automated workflow were predominantly sequenced on the NovaSeq 6000 platform because batch sizes could be increased, while most of the manually prepared libraries were sequenced on the NextSeq 500. The same number of read counts and target coverage of 1000× per sample were achieved for both sequencing platforms.

### Statistical Analysis

Statistical analysis was performed and the graphics generated using the statistical programming language R version 4.1.2 (R Foundation for Statistical Computing, Vienna, Austria) and the R package ggplot2 (version 3.4.4), respectively.^13^ Two-sided Wilcoxon test and two-sided paired Wilcoxon test were used to compare library performance data between the manual TSO 500 and the automated TSO 500 HT workflows.

## Results

### Validation Results

**Figure 1:**
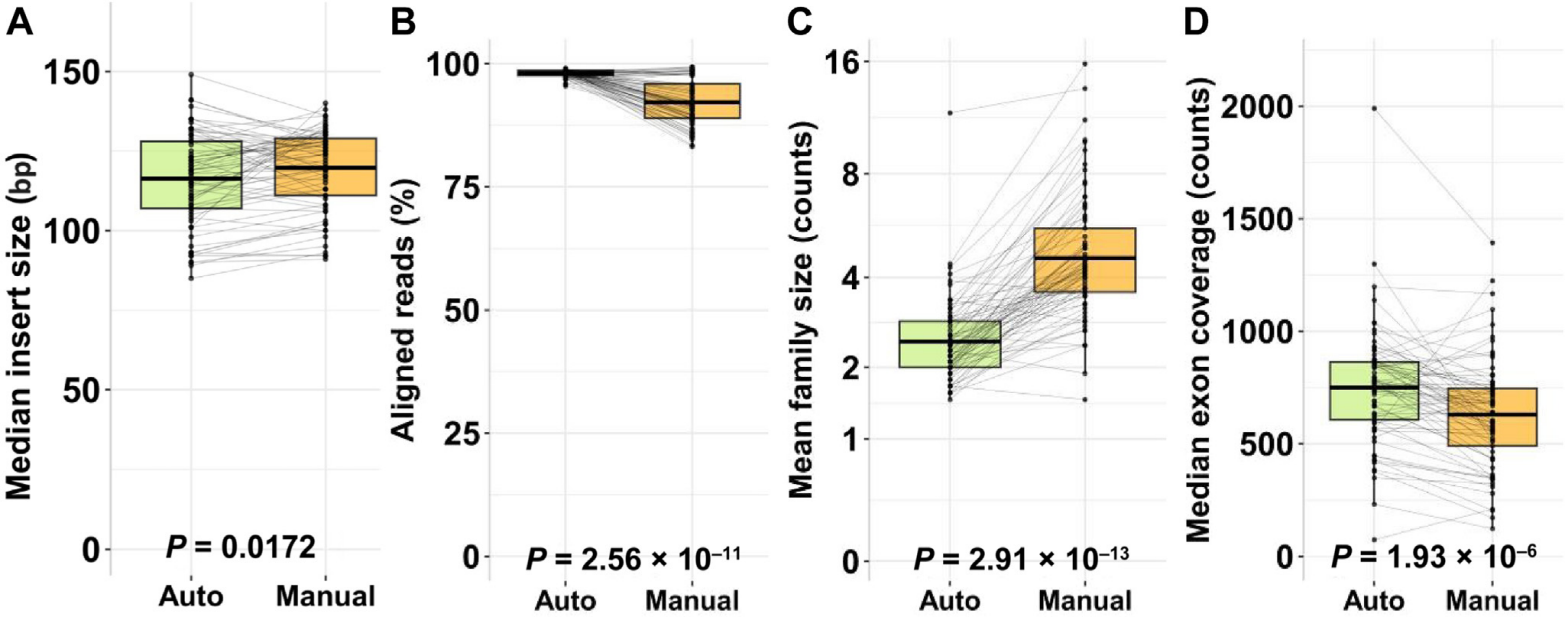
Validation of 73 samples in automated and manual workflows in terms of median insert size (**A**), aligned reads (**B**), mean family size (**C**), and median exon coverage (**D**).

For validation of the automated TSO 500 DNA HT workflow in combination with the Biomek i7, a direct 1:1 comparison of the automated versus manual workflows was performed using a validation cohort comprising 73 samples. A small but significant difference was observed regarding the median insert sizes of the automated workflow (116 bp) compared with the manual workflow (120 bp) (*P* = 0.0172) (Figure 1A), in line with the change in target fragmentation size. The percent aligned reads to the reference genome was high in both workflows but significantly lower at around 92% in the manual workflow versus almost 98% in the automated workflow (*P* = 2.1 × 10^−13^) (Figure 1B). The mean family sizes were significantly lower in the automated workflow, indicating a higher complexity of the generated libraries (*P* = 4.25 × 10^−17^) (Figure 1C); the result was slightly but significantly higher median exon coverage of 751 compared with 629 for the manual approach (Figure 1D).

**Figure 2:**
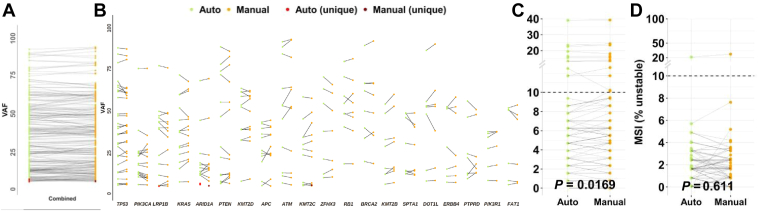
Validation of 73 samples in automated and manual workflows. The combined called mutations of the 20 most frequently occurring genes and their variant allele fraction (VAF) changes between the manual and automated workflow are shown combined (**A**) and separated by the gene of occurrence (**B**), as well as the complex biomarkers of total tumor mutational burden (**C**) and microsatellite instability (MSI) (**D**). The dashed lines in (**C**) and (**D**) represent the clinical cutoff for tumor mutational burden–high and MSI-high status, respectively.

Mutation calls with a variant allele fraction (VAF) cutoff at 3% were compared. VAFs of the mutation calls of the automated workflow were intersected with the previously known mutations of the manual workflow. The 20 most frequently altered genes in both workflows are shown in Figure 2. Of the initial 217 variants detected in the manual approach, 214 (98.6%) could also be identified in the automated workflow. Manual revision of the three missed variants (2× *ARID1A*, 1× *KMT2C*) revealed their presence in the alignment files of the automated workflow, slightly below the 3% threshold. VAF variances in general were within the expected range of resequencing of a sample (*P* = 0.416) (Figure 2, A and B). There were three additional mutation calls in the automated workflow (1× *LRP1B*, 2× *ARID1A*) that were not detected in the manual approach. Again, this discrepancy could be solved by manual review of the alignment files in which the variants were present with VAFs <3%.

Next, the clinically relevant biomarkers TMB and MSI were compared. In a paired comparison, the differences between the TMB values were significant (*P* = 0.0169), with a mean deviation of 0.63 mutation/Mb and a maximum of 2.33 mutations/Mb, which corresponds to less than one and three mutation calls, respectively. Manual revision of the highest deviating sample revealed three mutations with VAFs ranging from 6.2% to 8.2% in the manual workflow; these were also present in the automated workflow but fell below the 5% cutoff for TMB calculation with VAFs of 3.8% to 4.1%. This case was the only one in which the difference in the TMB value (manual, 10.15 mutations/Mb; automated, 7.82 mutations/Mb) would have led to a change in the TMB state (Figure 2C). There were no significant differences observed regarding the percentage of unstable MSI sites (*P* = 0.611) (Figure 2D).

### Clinical Cohort Comparison

**Figure 3:**
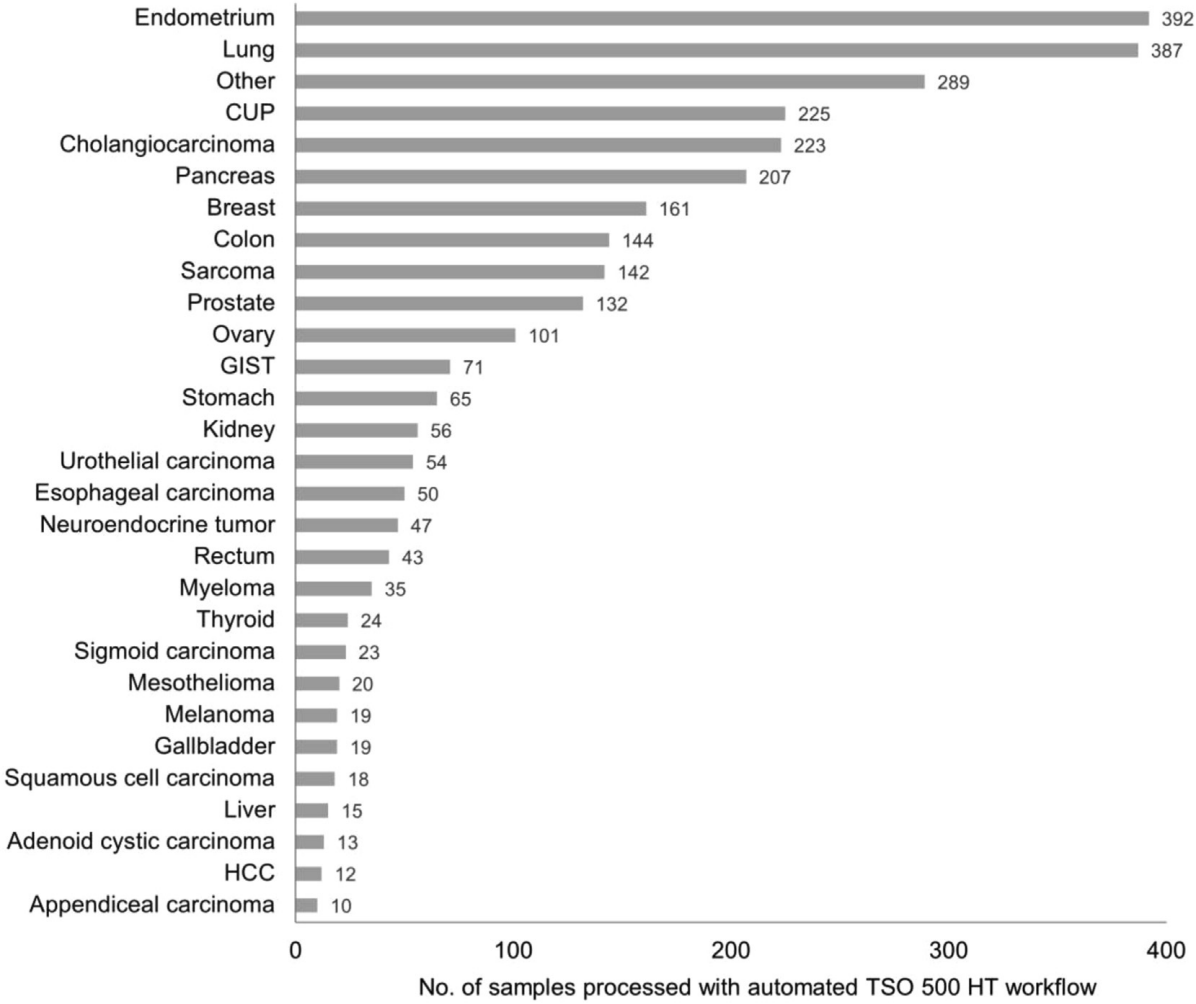
Tumor formalin-fixed, paraffin-embedded samples sequenced using the automated TruSight Oncology 500 DNA High Throughput (TSO 500 HT) assay (*n* = 2997). CUP, cancers of unknown primary; GIST, gastrointestinal stromal tumor; HCC, hepatocellular carcinoma.

A total of 2997 tumor samples were analyzed with the TSO 500 DNA HT assay (Figure 3) after introduction of automated library preparation using the Biomek i7 for clinical diagnostics. The distribution of samples revealed a predominant representation of endometrial and lung tumors (each approximately 13% of the samples). In addition, a substantial proportion of the samples (7.5%) were cancers of unknown primary because there is a clinical program on patients with these cancers. In contrast, liver tumors, adenoid cystic carcinoma, hepatocellular carcinoma, and appendiceal carcinoma exhibited lower sample counts. Notably, a total of 289 samples were combined in the “other” category for rare tumor types.

**Figure 4:**
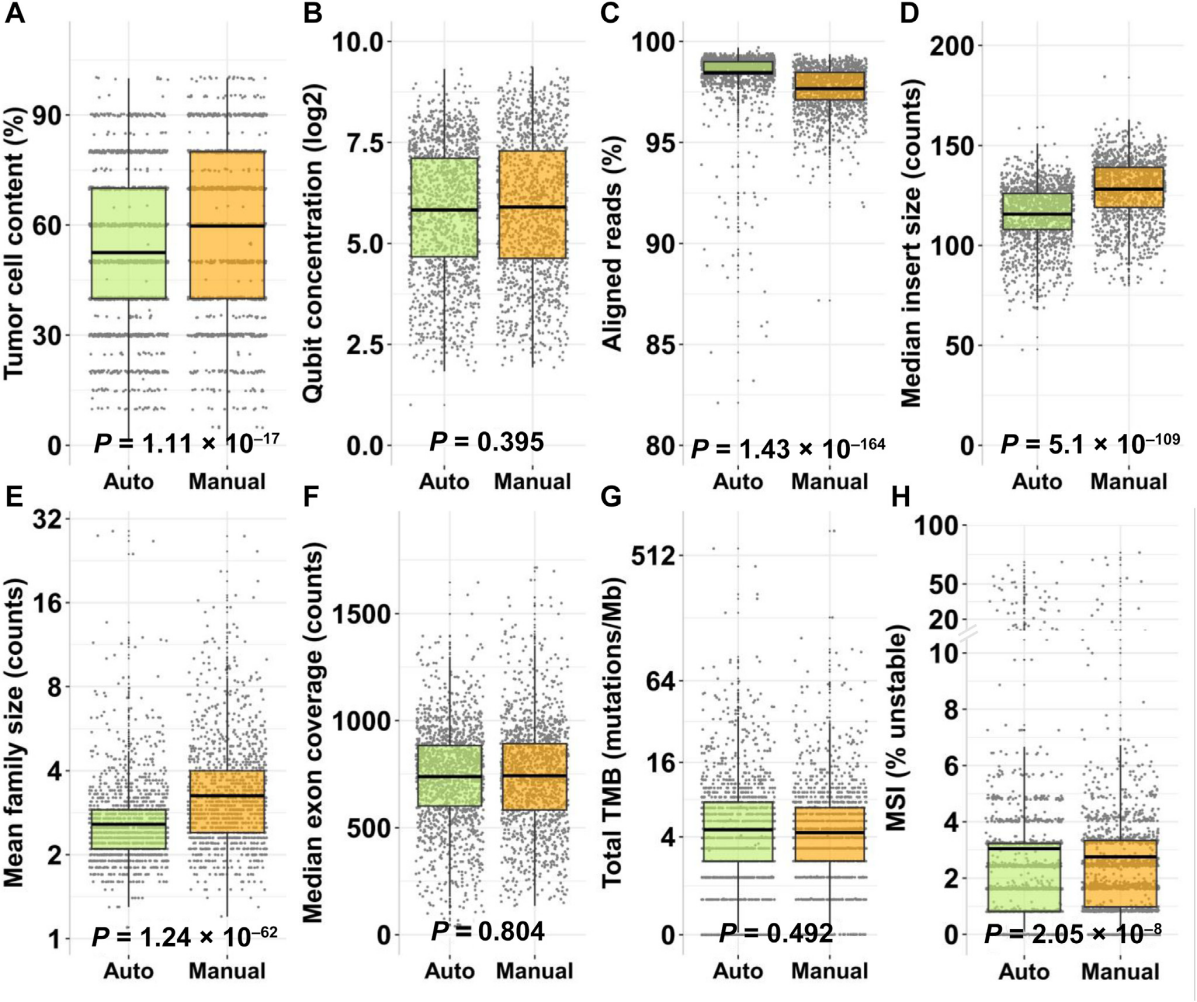
Performance comparison between automated and manual workflows in terms of tumor cell content (**A**), Qubit concentration (**B**), percentage of aligned reads (**C**), median insert size (**D**), mean family size (**E**), median exon coverage (**F**), total tumor mutational burden (TMB) (**G**), and microsatellite instability (MSI) (**H**).

For a performance evaluation of the manual TSO 500 DNA workflow (manual) versus the automated TSO 500 DNA HT workflow (automated) in the clinical setting, the automated cohort (2997 samples) was compared versus the previously sequenced manual cohort (2876 samples) (Figure 4). Quality control metrics of tumor cell content, sample concentration, aligned reads, median insert size, mean family size, median exon coverage, and the median of the biomarkers TMB and MSI were assessed. In the presequencing metrics, no significant differences in DNA concentrations were observed (*P* = 0.4), whereas the median tumor cell content was higher in the manual cohort (60%) than in the automated cohort (52%) (*P* = 1.1 × 10^−17^) (Figure 4, A and B).

**Figure 5:**
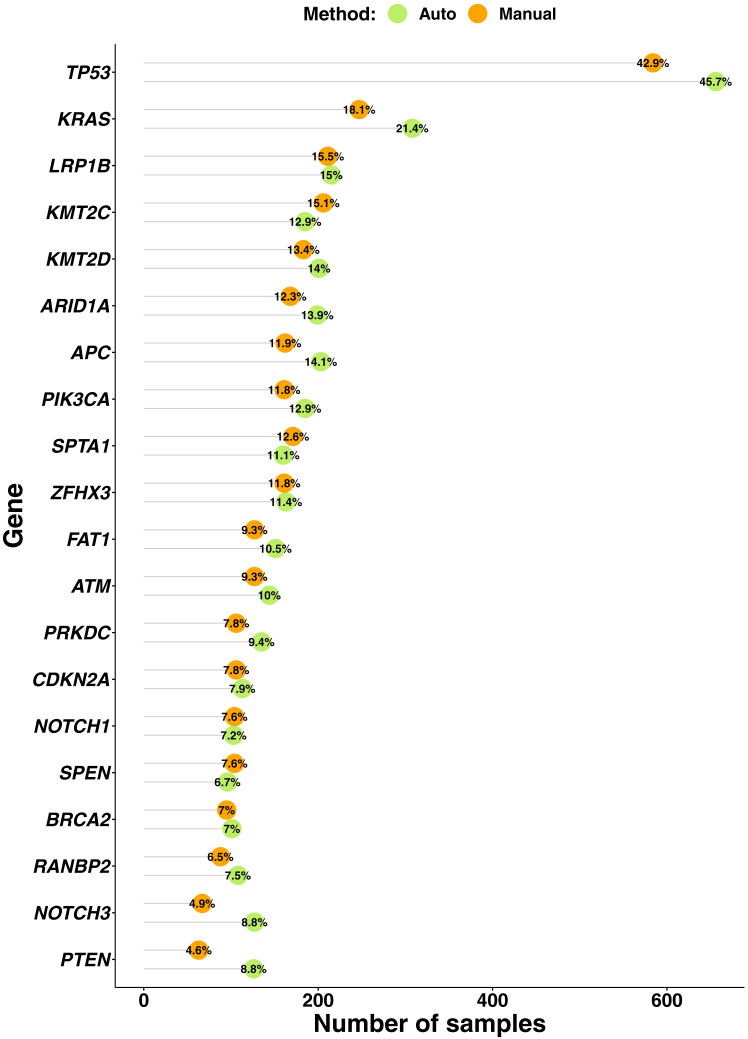
Analysis of the most frequently occurring alterations. Green dots indicate automated workflow, and orange dots indicate manual workflow.

Also, the automated workflow exhibited a slightly higher percentage of aligned reads (0.94%) (Figure 4C) and a 13 bp smaller median insert size (Figure 4D) compared with the manual workflow, showing a similar trend to the validation data set (Figure 1, A and B). The postsequencing quality control metrics showed a slight 0.6 count reduction of mean family size (Figure 4E) for the automated workflow, similar to the validation cohort. No significant difference was observed between the median coverage of both cohorts (Figure 4F). When comparing the biomarker results, the distribution of TMB values showed no significant difference between the two workflows (Figure 4G), whereas a small decrease in the percentage of MSI values was observed in the cohort of the automated workflow (Figure 4H). The comparison of the most frequently occurring alterations detected with each workflow showed that the most frequently altered gene was *TP53* in both cohorts (automated, 45.9%; manual, 42.9%) (Figure 5). Similar percentages were observed across other genes between both TSO 500 DNA workflow cohorts.

**Figure 6:**
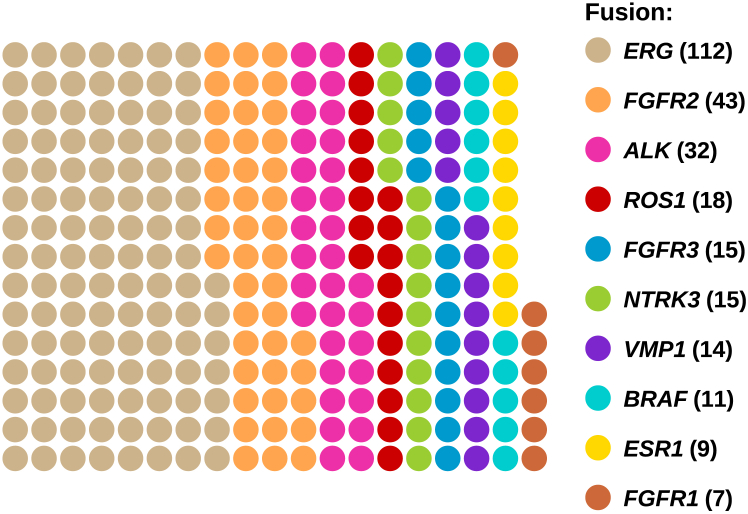
Waffle chart of number of samples with gene fusions.

With the introduction of the automated workflow into diagnostic testing, the TSO 500 RNA HT assay was added as a paired DNA/RNA analysis for a subset of samples. A total of 1127 RNA libraries were generated, with most of the RNA libraries sequenced together with their respective DNA sample. The 276 most frequently observed fusions are shown in Figure 6. The majority were *ERG* fusions (*n* = 112), followed by *FGFR2* (*n* = 43) and *ALK* (*n* = 32). Between 18 and 11 fusions were detected in *ROS1*, *FGFR3, NTRK3, VMP1*, and *BRAF*. *ESR1* and *FGFR1* were detected in lower numbers (9 and 7, respectively).

Finally, the runtime and hands-on time between the automated TSO 500 HT RNA and DNA library preparation on Biomek i7 hyb versus the manual workflow (Table 1) were compared considering a 16 DNA + 16 RNA sample batch and a 48 DNA + 48 RNA batch. The automated workflow required one technician regardless of the batch size, whereas one and three technicians would be necessary to prepare the same number of libraries manually, respectively. Interaction with the Biomek i7 hyb required only 1 to 2 hours per processing day (Table 1). Workflow automation resulted in a 4-fold reduction (6 hours vs. 23 hours) in hands-on time and a 1.7-fold reduction (24 hours vs. 42.5 hours) in total runtime compared with the manual library preparation of a 48 DNA + 48 RNA sample batch. For a 16 DNA and 16 RNA sample batch, while automation added 1 hour to the total runtime (23 hours vs. 22 hours), it reduced the hands-on time by 3 hours compared with manual library preparation (5 hours vs. 8 hours).

**Table 1:** Comparison of Automated TSO 500 HT RNA and DNA Library Preparation on Biomek i7 versus Manual Workflow

Step	Automated		Manual	
	16 RNA +16 DNA, min	48 RNA +48 DNA, min	16 RNA +16 DNA, min	48 RNA +48 DNA, min
Initialize Biomek i7, thaw reagents, cDNA synthesis (if RNA is processed)				
Hands-on time	45	60	100	300
Biomek i7 runtime	165	215	NA	NA
Total processing time	210	275	165	495
Library preparation∗ + setup Biomek i7				
Hands-on time	45	60	100	300
Biomek i7 runtime	180	235	NA	NA
Total processing time	225	295	180	540
Setup index PCR + first hybridization				
Hands-on time	35	50	50	150
Biomek i7 runtime	70	90	NA	NA
Total processing time	105	140	90	270
Shut down Biomek i7 (external first hybridization)				
Hands-on time	20	25	NA	NA
Biomek i7 runtime	Overnight	Overnight	Overnight	Overnight
Total processing time	Overnight	Overnight	Overnight	Overnight
Prep Biomek i7 first capture + second hybridization				
Hands-on time	45	60	110	330
Biomek i7 runtime	230	300	NA	NA
Total processing time	275	360	255	765
Prep Biomek i7 second capture + amplify enriched library				
Hands-on time	35	45	45	135
Biomek i7 runtime	125	160	NA	NA
Total processing time	160	205	110	330
Cleanup enriched amplified libraries				
Hands-on time	35	45	50	150
Biomek i7 runtime	40	50	NA	NA
Total processing time	75	95	50	150
Clean + shut down Biomek i7				
Hands-on time	35	45	NA	NA
Total processing time	35	45	NA	NA
Total technician hands-on time, min	295	384	455	1365
Total runtime, min	1105	1440	850	2550
No. of technicians†	1	1	1	3

NA, not applicable; TSO 500 HT, TruSight Oncology 500 DNA High Throughput.

∗ Start DNA only workflow.

† Number of technicians to prepare maximum recommended number of libraries.

Taken together, these results show that implementation of the automated TSO 500 HT workflow reduced hands-on time for batches of 16 DNA + 16 RNA samples and significantly lowered it to less than one third for 48 DNA + 48 RNA samples batches compared with manual library preparation. It maintained comparable or achieved even better quality control metrics for the DNA workflow.

## Discussion

Automation of sequencing workflows can help standardize high-volume sample processing to support the increasing demand for precision medicine. Library preparation for CGP is a multistep process with several hands-on phases. We show how automation of the TSO 500 DNA HT library preparation workflow achieved the same analytical quality as the manual workflow while significantly reducing hands-on time and processing times for large sample batches, also giving the possibility for including the TSO 500 RNA HT assay in the automated workflow. For smaller sample batches, although the overall processing time was similar to that of the manual workflow, the hands-on time was still significantly reduced, thus freeing up laboratory technicians’ time. Setting up the Biomek i7 hyb Workstation using the preconfigured deck layout for TSO 500 HT took only 1 to 2 hours each processing day, with the instrument then working autonomously until the next touch point. In addition, reduced manual interventions can also potentially decrease the risk of human-introduced errors.

Diagnostic laboratories processing a high volume of samples can greatly benefit from the automated protocol to reduce labor and operating costs associated with increased testing capacity. One technician ran the automated workflow for a 48 RNA + 48 DNA sample batch, while triple the personnel were needed to manually process the same number of samples. Given a steady high throughput of clinical samples, batch automation can facilitate planning of reagents and consumables for cost-effective laboratory management.

The head-to-head performance comparison between the automated workflow and the manual workflow validated the use in routine diagnostics. This was shown by the results of total TMB, percentage of MSI, and VAFs of recalled mutations in the most frequently altered genes. Discrepancies observed in the VAFs for mutations that were not found in the respective other sample were explainable upon manual review.

For the clinical cohort comparison, the automated workflow showed comparable performance to the manual workflow as indicated by presequencing and postsequencing metrics, including analysis of TMB and MSI and variant detection frequency. A slightly lower tumor cell content was observed in the automated sample set collected more recently than the manual cohort.

The observed lower family sizes of the libraries, and the resulting slight increases in median coverages between workflows, can be attributed to changes in the DNA shearing protocols as a contributing factor. This was due to having more unique molecules in the preferred fragment size range for the library amplification.

Finally, the automated RNA library preparation workflow could be introduced in the automated workflow. The most frequently detected gene fusions using the automated RNA workflow involved *ERG, FGFR2*, and *ALK*, consistent with the prevalence reported for similar pan-cancer cohorts. Limitation of this study includes some minor changes in protocol for fragmentation, use of different Illumina sequencing instruments, and the lack of a suitable comparison cohort for the RNA part of the TSO 500 RNA workflow.

## Conclusions

Improving clinical implementation of CGP is key for patients with cancer to benefit from precision oncology. Turnaround time of CGP results is one barrier to overcome for the timely delivery of targeted therapy. In this study, we showed that implementation of a fully automated TSO 500 HT assay significantly reduced hands-on time and processing time per sample compared with manual DNA and RNA library preparation. CGP workflow automatization in diagnostic laboratories is therefore a strategic solution to accelerate delivery of targeted therapy to patients with cancer.

## Disclosure Statement

M.K. has received personal fees for speaker honoraria and support for attending meetings or travel from Veracyte Inc., outside the submitted work. O.N. reports personal fees from Novartis outside the submitted work. A.-L.V. reports personal fees from AstraZeneca outside the submitted work. M.A. received speaker honoraria from Boehringer Ingelheim. P.S. reports personal fees for speaker honoraria BMS; grants from BMS, AstraZeneca, and MSD; and personal fees for advisory board participation from BMS, AstraZeneca, and MSD, outside the submitted work. J.B. reports grants from German Cancer Aid and consulting from MSD, outside the submitted work. A.S. has received personal fees for advisory board participation from Agilent, Aignostics, Amgen, AstraZeneca, Bayer, BMS, Eli Lilly, Illumina, Incyte, Janssen, MSD, Novartis, Pfizer, Qlucore, Roche, Seagen, Takeda, and Thermo Fisher; and grants from Bayer, BMS, Chugai, and Incyte, outside the submitted work. D.K. reports personal fees for speaker honoraria from AstraZeneca and Pfizer and personal fees for advisory board participation from Bristol Myers Squibb, outside the submitted work.
